# Study of microRNAs in Gingival Crevicular Fluid as Periodontal Diseases Biomarkers: Systematic Review

**DOI:** 10.3390/ijms25158274

**Published:** 2024-07-29

**Authors:** María Cosín-Villanueva, Pedro J. Almiñana-Pastor, Jose Luis García-Giménez, Andrés López-Roldán

**Affiliations:** 1 Department of Stomatology, Faculty of Medicine and Odontology, University of Valencia, 46010 Valencia, Spain; mariacosin27@gmail.com (M.C.-V.); andres.lopez-roldan@uv.es (A.L.-R.); 2 Biomedical Research Institute INCLIVA, 46010 Valencia, Spain; j.luis.garcia@epidisease.com; 3 Consortium Center for Biomedical Network Research on Rare Diseases, CIBERER-ISCIII, 46010 Valencia, Spain; 4 Department of Physiology, Faculty of Medicine and Odontology, University of Valencia, 46010 Valencia, Spain

**Keywords:** microRNA, periodontal diseases, periodontitis, epigenetic

## Abstract

Aim: The aim of this review was to identify the microRNAs (miRNAs) present in gingival crevicular fluid (GCF) that can be used as biomarkers for the diagnosis of periodontal diseases, and to determine which of them has a higher diagnostic yield for periodontitis. Methods: The review was conducted following the Preferred Reporting Items for Systematic Reviews and Meta-Analysis guidelines (reference number CRD42024544648). The Pubmed, Scopus, Cochrane Library, Embase, Web of Science, and Google Scholar databases were searched for clinical studies conducted in humans investigating periodontal diseases and miRNAs in GCF. The methodological quality of the articles was measured with the Newcastle–Ottawa Scale. Results: A total of 3222 references were identified in the initial literature search, and 16 articles were finally included in the review. The design of the studies was heterogeneous, which prevented a meta-analysis of the data. Most of the studies compared miRNA expression levels between patients with periodontitis and healthy controls. The most widely researched miRNA in periodontal diseases was miR-200b-3p and miR-146a. Conclusions: the miRNAs most studied are miR-146a, miR-200b, miR-223, miR-23a, and miR-203, and all of them except miR-203 have an acceptable diagnostic plausibility for periodontitis.

## 1. Introduction

Periodontitis is a chronic, multifactorial, and immunoinflammatory disease of microbial etiology, characterized by the destruction of the supporting dental tissues, which can lead to tooth loss [1].

The main etiological factor is the bacteria living in the biofilm [2]. A biofilm dysbiosis initiates the innate immune response which, in an effort to protect the organism, can in some individuals produce an exaggerated inflammatory immune response. This response can contribute to establishing a chronic low-grade inflammatory environment over long periods, thus contributing to the pathogenesis of periodontitis [2,3].

This is why inflammation and the immune response play a fundamental role in the pathogenesis of periodontitis. Any change in gene expression can alter the responses to inflammation and immunity. The process by which gene expression is modified without altering the DNA sequence is known as epigenetics [2].

Epigenetics includes processes such as DNA methylation, chromatin remodeling, histones post-translational modification, and non-coding RNAs, such as microRNAs (miRNAs) [2].

MiRNAs are short non-coding RNAs (19–24 nucleotides in length) that function through translational inhibition or mRNA destabilization via specific sequence binding sites within the 3′ untranslated region (UTR) of genes [4]. They regulate post-transcriptional gene expression, thereby influencing many biological processes in human cells that depend on DNA transcription and protein synthesis [2].

MiRNAs are considered an epigenetic mechanism that regulates gene expression and consequently modulates various cellular processes, such as cell growth, apoptosis, and cellular differentiation. Additionally, miRNAs play a fundamental role in inflammatory responses and in the development of diseases [5].

Systemic diseases such as cardiovascular diseases (CVD) [6], diabetes mellitus (DM) [3,7,8], obesity [9], and rheumatoid arthritis (RA) [10,11], all characterized by low-grade inflammation similar to that produced in periodontitis, are associated with alterations in miRNA levels.

Currently, clinical parameters such as clinical attachment loss (CAL), probing pocket depth (PPD), the presence and extent of angular bone defects, and the presence of furcation lesions, among others, are used to evaluate periodontal diseases. These parameters are aimed at determining the treatment and prognosis of the pathology. Therefore, their utility is limited for establishing an early diagnosis. Hence, identifying biomarkers that support the early diagnosis of periodontal diseases could be useful in reducing its incidence [12,13].

The most notable therapeutic importance of using a biomarker such as miRNA lies in its potential to detect periodontitis at an early stage, when periodontal tissue lesions are not yet clinically detectable. Consequently, this allows for early treatment of the disease to halt its progression before periodontal lesions are established [14].

When miRNAs are overexpressed or underexpressed, they can lead to changes in gene expression that may contribute to the onset and/or clinical progression of periodontitis [2]. Dysregulation of miRNAs can be detected in various bio-fluids such as serum, saliva, urine, gingival crevicular fluid (GCF), or cerebrospinal fluid. This makes them ideal candidates as specific and sensitive biomarkers for the diagnosis and prognosis of many diseases [15,16].

The reason biofluids were proposed as excellent sources for miRNA biomarker research includes the ease of isolation and identification of miRNAs using quantitative polymerase chain reaction (qPCR), the less invasive nature of miRNA sample collection, and their high stability in various biofluids [17].

In the context of periodontium, the most important biofluid is GCF. It flows passively from the gingival sulcus at the interface between the tooth and adjacent gum tissue. The volume of GCF increases and its composition alters according to the inflammatory status or disease of a specific periodontal site [18,19].

In recent years, numerous studies were published on the relationship between miRNAs and periodontal diseases, yet there is considerable heterogeneity in the literature regarding the miRNAs studied. The objective of this systematic review was to identify and summarize which miRNAs present in human GCF can be used as biomarkers for the diagnosis of periodontal diseases, and to determine which of them show the highest diagnostic performance for periodontitis.

## 2. Materials and Methods

The present review followed the guidelines of the Preferred Reporting Items for Systematic Reviews and Meta-Analysis (PRISMA) 2020 statement [20], whose item compliance list can be seen in Table S2. The protocol was registered in PROSPERO under ID: CRD42024544648. Available at: https://www.crd.york.ac.uk/prospero/display_record.php?ID=CRD42013004648 (accessed on 23 July 2024)

The Population, Exposition, Comparison, Outcome (PECO) question [21] formulated was as follows: Population (humans), Exposure (miRNAs expression in GCF), Comparison (healthy individuals), and Outcome (diagnosis of periodontal diseases). Therefore, the research question to be addressed was: What are the miRNAs present in human GCF that can be used as diagnostic biomarkers to differentiate subjects with periodontal disease from healthy individuals?

The inclusion criteria were cross-sectional, case-control, cohort, or experimental studies conducted in humans; using samples of GCF; addressing periodontitis and gingivitis; and analyzing miRNAs.

Regarding the exclusion criteria, studies lacking a control group or in vitro experimentation studies were excluded.

To retrieve all relevant studies that addressed the PECO question formulated, a rigorous search was conducted on 26 January 2024, across the following databases: PubMed, Scopus, Web of Science, Embase, Cochrane Library, and Google Scholar. No language or time limits were applied. The search was updated on 30 May 2024.

Additionally, a manual search was conducted on published systematic reviews on the topic, and the reference lists of articles used in this review were consulted.

To formulate the search strategy, a terminological analysis was initially performed. The process of formulating the search equations and their application in each of the databases was carried out by a single researcher (M.C.-V.).

The generic search strategy used was as follows: (“MicroRNAs” OR “MicroRNA” OR “miRNA” OR “miRNAs” OR “Micro RNA” OR “Micro RNAs” OR “mi-RNA” OR “mi-RNAs” OR “Primary MicroRNA” OR “Primary miRNA” OR “pri-miRNA” OR “pri miRNA” OR “pre-miRNA” OR “pre miRNA” OR “stRNA” OR “Small Temporal RNA”) AND (“Periodontal disease” OR “Periodontal diseases” OR “Gingivitis” OR “Periodontitis” OR “Gingival” OR “Periodontal” OR “Parodontosis” OR “Parodontoses” OR “Pyorrhea Alveolaris”).

This strategy was adapted for each of the databases consulted, as detailed in Table S1, aiming to strike a balance between precision and comprehensiveness. Therefore, the search was conducted by title and abstract, and using indexed terms in the MeSH thesaurus for PubMed and indexed terms in the Emtree thesaurus for Embase.

The article selection process, after removing duplicates, was independently conducted by two researchers (M.C.-V. and P.J.A.-P.) who evaluated the title and abstract. In cases of uncertainty, a third researcher (A.L.-R.) was consulted. Articles that could not be decisively rejected based on title and abstract were reviewed in full text.

Following this initial screening, all selected articles were read in full text to determine their inclusion or exclusion. Reasons for exclusion were noted for rejected articles.

The quality of observational studies included in the review was assessed using the Newcastle–Ottawa Quality Assessment Scale (NOS) for case-control studies. This scale, developed by Wells et al. in 2000 [22], evaluates eight items to determine the methodological quality of the study under analysis. These items are divided into three categories: selection, comparability, and exposure/outcome. Each observational study could score a maximum of nine points.

The quality assessment was conducted independently by two researchers (M.C.-V. and P.J.A.-P.), with a third researcher (A.L.-R.) consulted in case of uncertainty.

From each article, the following data were extracted: author and year of publication, country, study type, sample size, age, type of periodontal diseases studied, and the classification used, miRNAs studied, and outcomes (miRNA expression levels, area under the curve, and sensitivity and specificity as a diagnostic tool).

Data extraction was also independently performed by two researchers (M.C.-V. and P.J.A.-P.), with a third researcher (A.L.-R.) consulted in case of uncertainty.

The results were collected and synthesized in different Excel tables based on the objectives set to facilitate the comparison and analysis of the various extracted data.

## 3. Results

### 3.1. Study Selection

The search strategy was conducted across six different databases, yielding: 581 documents in PubMed, 831 in Embase, 845 in Scopus, 876 in Web of Science, 12 in Cochrane Library, and 77 in Google Scholar. In total, 3222 documents were retrieved, and after removing duplicates (*n* = 2091) across databases, 1131 remained for title and abstract screening.

Manual searching did not yield any articles not previously retrieved through electronic search.

Initial screening after title and abstract review excluded 1106 documents. Most were excluded because they were literature reviews or editorials (*n* = 359), in vitro or animal studies (*n* = 445), not related to the research question (*n* = 15), studying other genetic mechanisms or pathologies (*n* = 190), retracted studies (*n* = 7), or investigating miRNA expression in samples other than GCF (*n* = 90).

Twenty-five articles met the inclusion criteria or lacked sufficient information in the title and abstract for rejection. Upon full-text reading, nine articles were excluded (Table S4). Reasons for exclusion were review article (*n* = 2), duplicate publication (*n* = 1), in vitro experimental study (*n* = 3), and investigating miRNA expression in samples other than GCF (*n* = 3).

**Figure 1:**
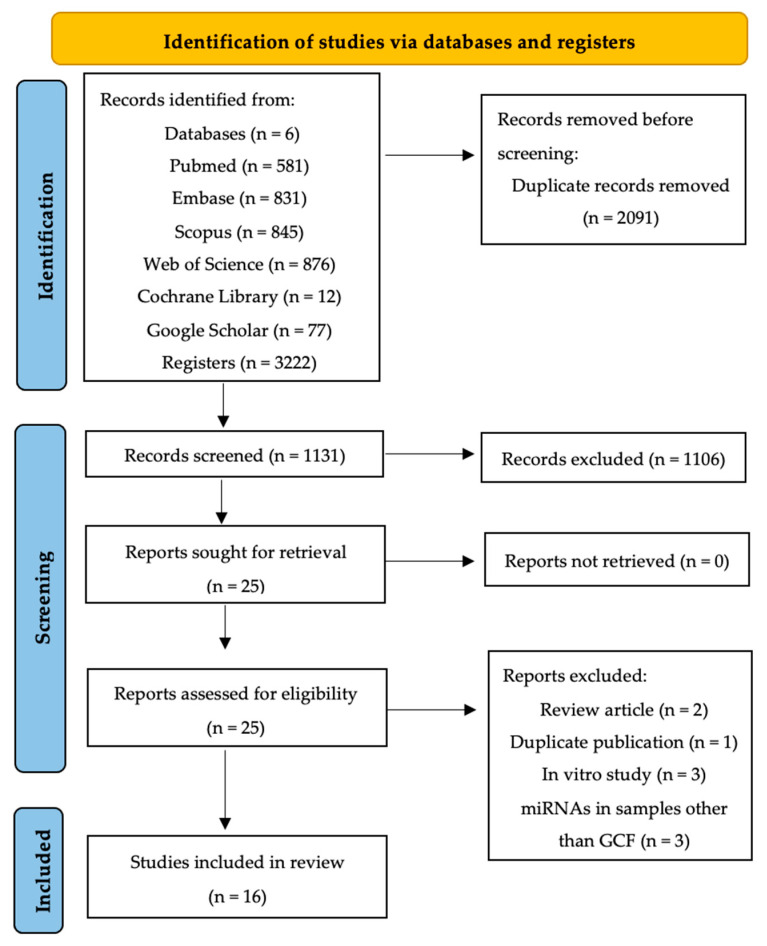
PRISMA flow diagram of the search process across the different databases.

Therefore, sixteen articles were finally included in the qualitative review. The PRISMA flow diagram (Figure 1) summarizes the article selection process.

### 3.2. Quality Assessment

Quality assessment for observational studies is detailed in Table 1. Overall, the quality assessment showed positive outcomes, as 13 out of the 16 included studies demonstrated good quality. Among the three studies that showed fair quality [13,23,24], biases mainly stemmed from not specifying the source of cases and/or controls, inadequate control of confounding factors, and failure to specify the non-response rate.

### 3.3. Characteristics of Included Studies

The characteristics of the studies included in the review are summarized in Table 2. Of the sixteen selected studies, all were observational case-control studies. Sample sizes varied across studies, ranging from 18 [31] to 216 [23] patients, with mean ages ranging from 27.8 years [13] to 65.3 years [13].

Most studies compared miRNA expression levels between patients with periodontitis and healthy controls. However, some authors assessed the influence of systemic diseases such as DM [7,8], AR [11,28], or CVD [6] on relative miRNA expression levels.

The Classification of Periodontal and Peri-implant Diseases and Conditions 2018 [32] was used in two-thirds of the studies, while the other third used the Armitage classification [33,34].

Upon analyzing the miRNAs proposed in the various included studies, it was observed that the information was highly heterogeneous, with the most analyzed ones being:

- miR-146a [7,19,28]
- miR-200b family:
  − miR 200b [3,8]
  − miR-200b-3p [3,6,8,13,23]
  − miR 200b-5p [6,23]
- miR-223 family:
  − miR-223 [3,8,13,25,26,31,35]
  − miR-223-3p [13,25]
  − miR 223-5p [6,31]
- miR-23a [8,24,25]
- miR-203 [3,8,13]

In most of these studies, miRNA expression levels (Table S3) were found to be upregulated in patients with periodontal involvement. However, some studies reported contradictory results:

o miR-21-3p: was found to be overexpressed in one study [6] and underexpressed in another [13].
o miR-146a-5p: was found to be overexpressed in two studies [7,19] and underexpressed in another [28].
o miR-200a-5p: was found to be overexpressed in one study [23] and underexpressed in another [13].
o miR-200b-3p: showed no alteration in two studies [6,23] and underexpressed in another [13].
o miR-200b-5p: was found to be overexpressed in one study [23] and showed no alteration in another [13].
o miR-200c-3p: showed no alteration in one study [23] and underexpressed in another [13].
o miR-200c-5p: was found to be overexpressed in one study [23] and underexpressed in another [13].

In Table 3, a summary of the miRNAs that were shown to be over- or underexpressed according to the severity of periodontitis can be observed.

Additionally, some authors observed correlation between relative expression levels of specific miRNAs and disease severity, showing positive correlation for miR-200a, miR-200b and miR-200c [23]; miR-103a-3p, miR-423-5p [25]; miR-223 [8,26]; miR-30b-3p, miR-125b-1-3p [27], miR-140-3p, miR-145-5p [28], and miR-3198 [11]; no correlation for miR-15a-5p and miR-223-3p [25]; and negative correlation for miR-23a-3p [25], miR-146a-5p [28], and miR-28-5p [29].

Regarding the use of miRNAs as diagnostic biomarkers for periodontitis, the proposed miRNAs were miR-200b [3,8,23], miR-146a-5p [7,19,28], and miR-223 [3,8,26]. Table 4 illustrates the miRNAs described as diagnostic biomarkers with values of area under the curve (AUC), sensitivity, and specificity.

## 4. Discussion

In the etiopathogenesis of periodontitis, epigenetic mechanisms such as miRNAs appear to play a crucial role in inflammation regulation. Despite miRNAs being proposed years ago as biomarkers for periodontal diseases, there remains considerable variability among the miRNAs studied and the type of samples used. This review was conducted to identify the miRNAs described in the literature for the diagnosis of periodontal diseases, focusing on studies using GCF samples. This biofluid is considered easily accessible, minimally invasive, and closely related to the site of inflammation, namely the periodontium.

Due to the impossibility of conducting a data synthesis and meta-analysis, some items (13, 14, 20, 22, and 23b) of the Preferred Reporting Items for Systematic reviews and Meta-Analyses (PRISMA) 2020 statement [20] were not fulfilled.

### 4.1. Strengths and Weaknesses of the Studies

In the assessment of the quality of observational studies, selection bias was observed in two studies [23,24] due to two reasons: lack of representativeness of cases and failure to specify the origin of controls. This bias was mitigated by controlling for confounding factors and achieving comparable study groups.

The sample size of a study influences the reproducibility of results. Small sample sizes can lead to the generation of false positives or negatives due to the limited portion of the sampled population, as seen in Almiñana-Pastor et al. (2023) [19] with N = 23, Micó-Martínez et al. (2018) [31] with N = 18, and Saito et al. (2017) [13] with N = 20. Therefore, the validity of findings from these studies is compromised. Similarly, studies that showed disparities between healthy and diseased groups [28] also could not achieve the same validity, as one of the groups was underrepresented.

The disease classification alongside the selected miRNAs were two variables that exhibited significant variation across the studies, complicating interpretation and comparison of results. Most of the studies [3,6,8,11,25,26,27,28,29,36] utilized the current Classification of Periodontal and Peri-implant Diseases and Conditions 2018 [1], with a diversity in the stages selected, predominantly focusing on stages II, III, and IV [11,25,27,28], which could correspond to moderate/severe periodontitis in the older Armitage classification [33,34].

It is noteworthy that only Rovas et al. (2022) [28], Zhu and Zhong (2022) [27], and Yu (2023) [23] included subjects in stage I, indicating that only three studies considered individuals in the early stages of periodontitis—a critical focus for identifying disease markers. Subsequently, studies should examine extreme groups (healthy subjects and those with advanced disease). Additionally, no article considered comparing miRNA levels across different grades, which is essential, as it refers to disease progression: Grade A (low susceptibility to progression), Grade B (moderate susceptibility to progression), and Grade C (high susceptibility to progression).

Similarly, only two authors [7,24] measured miRNA levels basic periodontal treatment, confirming that miRNA levels decrease to physiological levels once inflammation is eliminated and the disease is controlled. This underscores the dynamic nature of these markers and their role in regulating cellular immune-inflammatory states.

### 4.2. MiRNA

In the search for miRNAs present in GCF, only Almiñana-Pastor et al. (2023) [19] conducted high-throughput sequencing, while the remaining authors focused on specific miRNA searches, potentially excluding miRNAs that could be considered biomarkers for periodontal diseases.

Among the most studied miRNAs was the miR-200b family, specifically miR-200b-3p. MiR-200b is involved in the differentiation of various immune cells, including macrophages, neutrophils, and others, and plays a significant role in the early stages of infection and inflammation [8]. In the vast majority of studies, miR-200b-3p levels were significantly elevated in GCF [3,6,8,23], although Saito et al. (2017) [13] reported lower levels, possibly due to their small sample size. Furthermore, miR-200b was associated with comorbidities such as periodontitis and type 2 DM [3,8], and periodontitis and CVD [6]. Elevated levels of miR-200b were also observed in gingival biopsies of subjects with obese periodontitis [37] and in gingival tissues [38]. These studies open new avenues for further research into the relationship between systemic diseases and periodontal diseases.

Both type 2 DM and CVD are chronic conditions that, similar to periodontitis, contribute to generating low-grade inflammation over long periods of time. Since miR-200b is involved in the initial stages of inflammation, the presence of not just one but two inflammatory pathologies will result in even higher miRNA levels in the presence of comorbidities. In the study by Liu et al. (2022) [8], the expression levels of miR-200b in the periodontitis group and the T2DM group were 3.06 ± 0.21 and 2.74 ± 0.22, respectively, while in the comorbid group, the levels were 3.51 ± 0.30, showing statistically significant differences compared to the other groups.

MiR-146a plays a role in various inflammatory processes and is involved in the activation of signaling pathways and cytokine secretion. It is also affected by pro-inflammatory stimuli, such as lipopolysaccharides from *Porphyromonas Gingivalis* (LPS), which, in response to bacterial stimuli, negatively regulates membrane receptor (TLR) signaling, favoring bacterial pathogenic action. MiR-146a was found to be upregulated in the studies by Almiñana-Pastor et al. (2023) [19] and Radović et al. (2018) [7]. In fact, in the latter study, miR-146a levels normalized after non-surgical periodontal treatment, indicating its expression is related to the inflammatory process of periodontitis.

However, different results were obtained in the study by Rovas et al. (2022) [27], where miR-146a levels were underexpressed in subjects with periodontitis compared to healthy subjects. The hypothesis suggested for these results is that negative regulation or impaired function of miR-146a may be associated with diseases characterized by sustained exaggerated inflammation, such as aggressive forms of periodontitis. Multiple studies analyzed miR-146a in gingival biopsies [39,40], saliva [41,42], or in vitro [43] and observed its overexpression in periodontitis. Therefore, further studies with larger samples in GCF are needed to conclusively determine whether miR-146a is under or overexpressed in periodontal diseases.

The overexpression of certain miRNAs, such as miR-23a, plays a destructive role in disease progression as it could inhibit osteogenesis [44]. Costantini et al. (2023) [25] observed statistically significant increases in levels of this miRNA in patients with mild-stage periodontitis compared to healthy subjects and moderate to severe stages. In the study by Zhang et al. (2019) [24], miR-23a significantly increased not only in GCF, but also in human periodontal ligament stem cell (PDLSC) cultures, inhibiting osteogenesis, and levels decreased significantly following periodontal therapy. This suggests that miR-23a may be elevated in all stages of the disease, playing a greater role in bone loss during the early phases of periodontitis. Therefore, studying its alteration in the subclinical stages of the disease would be interesting, as it may act as an initiator of bone metabolism disturbance.

One of the changes occurring in inflamed tissues is increased angiogenesis. In the case of periodontal diseases, miR-203 plays a crucial role in this change by targeting vascular endothelial growth factor A (VEGFA), thereby inhibiting angiogenesis [45]. Thus, underexpression of miR-203 could be associated with the presence of periodontitis. The three studies that included miR-203 among the studied miRNAs found it to be underexpressed [3,8,13]. In fact, Liu et al. (2022) [8] and Elazazy et al. (2021) [3] observed a negative correlation of miR-203 with TNF-α, explaining the decreased healing and suggesting its impact on irreversible damage caused by the disease. Similar results were seen in other studies conducted on gingival tissue [16,46]. Therefore, miR-203 could potentially play a protective role in periodontitis, not only for diagnosis, but also as a therapeutic target.

Another extensively studied miRNA was miR-223, which functions as a key regulator of innate immunity by influencing the differentiation of various immune cells [47] and is crucial in regulating osteoclast differentiation, thus impacting the pathological process of bone remodeling [48].

Two included studies [3,8] reported that miR-223 expression levels in GCF were higher in patients with periodontitis compared to healthy controls, with one of them showing statistically significant differences [8]. Both articles included subjects with periodontitis, with and without DM, and in both studies, miR-223 levels were much higher in subjects with periodontitis and DM compared to those with periodontitis alone. This finding holds promise for precision medicine, as GCF can reveal miRNAs associated with systemic pathologies. Similar results of overexpression were found in gingival tissue biopsies [37], in the serum of a mouse model with periodontitis [47], and in gingival crevicular blood [35].

There also appeared to be a positive correlation between miR-223 levels and disease severity, as determined by increased TNF-α and clinical parameters in the chronic periodontitis group compared to healthy controls [3]. However, in the study by Costantini et al. (2023) [25], miR-223-3p did not show correlation with the severity of periodontitis.

Given that for a diagnostic tool to be considered acceptable, it should have an AUC > 0.8 [49], among all the miRNAs proposed as diagnostic biomarkers, those that could truly be employed for the diagnosis of periodontitis were: miR-146a [7], miR-23a [24], miR-28-5p [29], miR-155 [7], miR-125b-1-3p [27], miR-200c-3p [23], miR-200b [8], miR-30b-3p [27], miR-200a-5p [23], miR-1226 [30], miR-200a-3p, miR-200b-5p [23], miR-223 [8], miR-223-5p [26], miR-200c-5p [23], and miR-200b-3p [23]. Therefore, the rest of the miRNAs proposed by authors as possible biomarkers for periodontitis, although they might have an AUC close to 0.8, would not achieve sufficient performance to be used as a diagnostic tool. Increasing the sample size could potentially change this result, hence, more well-designed studies are needed to analyze the diagnostic value of these miRNAs.

### 4.3. Limitations of the Review

The main limitation was the inability to perform a quantitative analysis of the data due to methodological differences among studies, particularly the discrepancies in the miRNAs studied across different studies, the absence of numerical data in publications, and the inability to obtain them through contact with authors. Additionally, different units of miRNA expression were used without the possibility of converting them into a common unit.

Another limitation was the lack of search in grey literature, which possibly resulted in selection bias by excluding relevant research from the review.

### 4.4. Future Recommendations

This systematic review identified the need for a more homogeneous approach within published studies on the identification of miRNAs in GCF in subjects with periodontitis:

- Following the miRNA high-throughput sequencing by Almiñana-Pastor et al. (2023) [19], and once the miRNAs involved in periodontitis are identified, efforts should focus on those that demonstrated differences between subjects with periodontal disease and those with healthy gingiva, to avoid costly comprehensive sequencing studies and to limit the selected sample.
- Employing the classification of Periodontal and Peri-implant Diseases and Conditions from 2018.
- Studying how miRNA expression levels vary among different grades of periodontitis as another item to establish disease progression.
- Standardizing the units of miRNA expression to enable quantitative data analysis, rather than limiting ourselves to the binary terms overexpressed and underexpressed.
- Conducting follow-up studies to re-measure miRNA levels after basic periodontal treatment.

## 5. Conclusions

MiRNAs were proposed as promising biomarkers for periodontal diseases in GCF, capable of reflecting the inflammatory regulation status of periodontal tissues at a given moment. Among the miRNAs that showed significant differences between individuals with periodontitis and periodontally healthy subjects are miR-146a, miR-200b (specifically miR-200b-3p), miR-223, miR-23a, and miR-203. These miRNAs, except for miR-203, exhibited acceptable diagnostic performance for use as diagnostic biomarkers. However, due to the substantial heterogeneity in the miRNAs studied, there is a need for studies with more homogeneous criteria that allow for quantitative synthesis to better assess their diagnostic utility.

## Figures and Tables

**Table 1:** Quality assessment of observational studies based on the Newcastle–Ottawa Quality Assessment Scale (NOS).

Case-Control Study	Selection				Comparability	Exposure			Total
	Case Definition *	Representativeness of the Cases *	Selection of Controls *	Definition of Controls *	The Groups Are Comparable, and the Most Important Confounding Factor Is Controlled *, as Well as Even other Factors **	Ascertainment of Exposure *	Same Method of Ascertainment for Cases and Controls *	Non-Response Rate *	
[19]	*	*		*	**	*	*		7/9
[23]	*			*	*	*	*		5/9
[25]	*	*	*	*	*	*	*		7/9
[26]	*	*	*	*	*	*	*		7/9
[6]	*	*	*	*	**	*	*	*	9/9
[8]	*	*		*	**	*	*		7/9
[27]	*	*	*	*	*	*	*		7/9
[28]	*	*	*	*	*	*	*		7/9
[29]	*	*	*	*	*	*	*		7/9
[30]	*	*	*	*	**	*	*		8/9
[11]	*	*	*	*	*	*	*		7/9
[3]	*	*	*	*	*	*	*		7/9
[24]	*			*	*	*	*		5/9
[31]	*	*	*	*	*	*	*		7/9
[13]	*	*	*	*		*	*		6/9
[7]	*	*	*	*	**	*	*		8/9

**Table 2:** Characteristics and main results of included studies.

Author (Year)	Country	Type of Study	N MA (Mean ± SD)	Periodontitis Classification	miRNA Studied	Results
Almiñana-Pastor et al. (2023) [19]	Spain	Case-control	N = 23 Case n = 11 MA (50.17 ± 7.34) Control n = 12 MA (46.36 ± 9.88)	Advance CP	High-throughput sequencing Select: miR-30a-5p miR-199b-3p miR-338-5p miR-146a-5p	Overexpression miR-30a-5p, miR-199b-3p, miR-338-5p and miR-146a-5p.
Yu (2023) [23]	Japan	Case-control	N = 216 Case n = 103 MA (46) Control = 113 MA (45)	Periodontitis stage I–IV	miR-200a-3p miR-200a-5p miR-200b-3p miR-200b-5p miR-200c-3p miR-200c-5p miR-141-3p miR-141-5p miR-429	Overexpression miR-200a-3p, miR-200a-5p, miR-200b-3p, miR-200b-5p, miR-200c-3p and miR-200c-5p
Costantini et al. (2023) [25]	Italy	Case-control	N = 36 Case n = 21 − Stage II n = 8 − Stage III n = 6 − Stage IV n = 7 MA (60.4 ± 3) Control n = 15 MA (58.5 ± 3.5)	Periodontitis stage II–IV	miR-103a-3p miR-23a-3p miR-15a-5p miR-223-3p miR-423-5p	Overexpression miR-103a-3p, miR-23a-3p, miR-15a-5p and miR-223-3p
Bandi et al. (2023) [26]	India	Case-control	N = 100 Case n = 50 MA (47.66 ± 11.2) Control = 50 MA (31.15 ± 8.39)	Periodontitis stage II grade B untreated	miR-223-5p	Overexpression miR-223-5p
Isola et al. (2022) [6]	Italy	Case-control	N = 115 Case n = 30 MA (53 (50.5–56) Case + CVD n = 29 MA (52 (51–55.5) Control n = 28 MA (52.5 (49–55.8) Control + CVD n = 28 MA (52.5 (51–56)	Periodontitis stage II grade C generalized	miR-7a-5p miR-21-3p miR-21-5p miR-100-5p miR-125-5p miR-200b-3p miR-200b-5p	Overexpression miR 7a-5p, miR 21-3p, miR-21-5p, miR-200b-3p and miR-200b-5p. Underexpression miR 100-5p and miR 125-5p
Liu and cols (2022) [8]	China	Case-control	N = 97 Case n = 26 MA (37.74 ± 6.25) Case + DM n = 22 MA (38.89 ± 6.35) Control n = 25 MA (39.66 ± 6.28) Control + DM n = 24 MA (40.15 ± 6.33)	Periodontitis stage II	miR-21c miR-22b miR-23a miR-103 miR-106 miR-141 miR-158 miR-201a miR-203 miR-200b miR-223	Overexpression miR-223 and miR-200b. Underexpression miR-203
Zhu and Zhong (2022) [27]	China	Case-control	N = 180 Case n = 80 − Stage I–II n = 44 − Stage III–IV n = 38 MA (40.53 ± 8.19) Control n = 100 MA (38.86 ± 7.77)	Periodontitis stage I–IV	miR-30b-3p, miR-125b-1	Overexpression miR-30b-3p and miR-125b-1-3p
Rovas et al. (2022) [28]	Lithuania	Case-control	N = 210 Case n = 78 Case + RA n = 56 MA (53.23 ± 10.44) Control n = 48 Control + RA n = 28 MA (46.04 ± 13.86)	Periodontitis stage I–IV	miR-140-3p miR-145-5p miR-146a-5p miR-195-5p	Overexpression miR-145-5p and underexpression miR-146a-5p
Huang and Jia (2022) [29]	China	Case-control	N = 147 Case n = 76 MA (47 ± 6) Control n = 71 MA (48 ± 8)	CP EFP/AAP classification	miR-28-5p	Underexpression miR-28-5p
Du et al. (2021) [30]	China	Case-control	N = 122 Case n = 72 MA (48.27 ± 6.99) Control n = 50 Ma (50.13 ± 6.70)	Moderate/ severe CP	miR-1226	Underexpression miR-1226
Rovas et al. (2021) [11]	Lithuania	Case-control	N = 61 Case n = 14 Case + RA = 16 MA (58.03 ± 6.84) Control n = 22 Control + RA = 9 MA (49.74 ± 13.31)	Periodontitis Stage II–IV	miR-199a-5p miR-483-5p miR-3198 miR-4299	Overexpression miR-3198 (1.9 times)
Elazazy et al. (2021) [3]	Egypt	Case-control	N = 60 Case n = 20 MA (40.45 ± 8.31) Case with DM n = 20 MA (48.47 ± 7.17) Control n = 20 MA (37.40 ± 6.13)	Periodontitis stage II	miR-223 miR-203 miR-200b	Overexpression miR-223 y miR-200b and underexpression miR-203.
Zhang et al. (2019) [24]	China	Case-control	N = 50 Case n = 29 MA (37.8 ± 2.0) Control n = 21 MA (41.5 ± 2.4)	Moderate/ severe periodontitis	miR-23a	Overexpression miR-23a
Micó-Martínez et al. (2018) [31]	Spain	Case-control	N = 18 Case n = 9 MA (50.44 ± 8.09) Control n = 9 MA (33.33 ± 12.05)	Moderate/ severe CP	miR-671 miR-122 miR-1306 miR-27a miR-223 miR-1226-5p	Underexpression miR-27a and miR-1226-5p (15.8 times). miR-671 and miR-122 were not detected; and miR-1306 and miR-223 showed no variation
Radović et al. (2018) [7]	Serbia	Case-control	N = 96 Case n = 24 Case + DM n = 24 Control n = 24 Control + DM n = 24 MA (44.13 ± 12.46)	Moderate/ severe CP	miR-146a miR-155	Overexpression miR-146a and miR-155
Saito et al. (2017) [13]	Japan	Case-control	N = 20 Case n = 9 Control n = 11 MA (27.8–65.3)	Moderate/ severe CP	Sequencing 619 miRNAs Select 40 miRNAs	Overexpression miR-101-3p; miR 126-5p; miR 140-5p; miR-144-3p; miR-144-5p; miR-145-5p; miR-194-5p; miR-19a-3p; miR-20a-3p; miR-20a-5p; miR-223-3p; miR-26b-5p; miR-301a-3p; miR-30e-3p; miR-30e-5p; mir 338-3p; miR-374a-5p; miR-582-5p and miR-590-5p Underexpression let-7e-5p; let-7i-5p; miR-100-5p; miR-125a-5p; miR-125b-5p; miR-155-5p; miR-181b-5p; miR-187-3p; miR-200a-5p; miR-200b-3p; miR-2006-5p; miR-200c-3p; miR-200c-5p; miR-203a; miR-205-5p; miR-21-3p; miR-210-3p; miR-222-3p; miR-31-3p; miR-320a and miR-99a-5p

SD: standard deviation; MA: main age; CVD: cardiovascular disease; DM: diabetes mellitus; RA: rheumatoid arthritis; miR: microARN; CP: chronic periodontitis; and EFP/AAP: European Federation of Periodontology/American Academy of Periodontology.

**Table 3:** Over- or underexpressed miRNAs according to the severity of periodontitis.

Periodontitis	Overexpressed miRNA	Underexpressed miRNA
Stage I or mild	miR-200a-3p [23], miR-200a-5p [23], miR-200b-3p [23], miR-200b-5p [23], miR-200c-3p [23], miR-200c-5p [23], miR-30b-3p [27], miR-125b-1-3p [27], miR-145-5p [28]	miR-146a-5p [28]
Stage II or moderate	miR-223-5p [26], miR 7a-5p [6], miR 21-3p [6], miR-21-5p [6], miR-200b-3p [6], miR-200b-5p [6], miR-223 [3,8], miR-200b [3,8], miR-23a [24], miR-146a [7], miR-155 [7]	miR 100-5p [6], miR 125-5p [6], miR-203 [3,8], miR-27a [31], miR-1226-5p [31]
Stage II–IV or moderate/severe	miR-200a-3p [23], miR-200a-5p [23], miR-200b-3p [23], miR-200b-5p [23], miR-200c-3p [23], miR-200c-5p [23], miR-103a-3p [25], miR-23a-3p [25], miR-15a-5p [25], miR-30b-3p [27], miR-125b-1-3p [27], miR-101-3p [13], miR 126-5p [13], miR 140-5p [13], miR-144-3p [13], miR-144-5p [13], miR-194-5p [13], miR-19a-3p [13], miR-20a-3p [13], miR-20a-5p [13], miR-26b-5p [13], miR-301a-3p [13], miR-30e-3p [13], miR-30e-5p [13], mir 338-3p [13], miR-374a-5p [13], miR-582-5p [13], miR-590-5p [13], miR-223-3p [13,25], miR-145-5p [13,28]	miR-146a-5p [28], miR-1226 [11], miR-27a [31], miR-1226-5p [31], let-7e-5p [13], let-7i-5p [13], miR-100-5p [13], miR-125a-5p [13], miR-125b-5p [13], miR-155-5p [13], miR-181b-5p [13], miR-187-3p [13], miR-200a-5p [13], miR-200b-3p [13], miR-2006-5p [13], miR-200c-3p [13], miR-200c-5p [13], miR-203a [13], miR-205-5p; miR-21-3p [13], miR-210-3p [13], miR-222-3p [13], miR-31-3p [13], miR-320a [13], miR-99a-5p [13]
Stage III and IV or severe	miR-30a-5p [19], miR-199b-3p [19], miR-338-5p [19], miR-146a-5p [19]	

miR: microRNA.

**Table 4:** Diagnostic performance of miRNAs used in included studies.

	N	AUC	Sensitivity (%)	Specificity (%)
miR-146a [7]	96	0.999	100%	96%
miR-200a-3p, miR-200a-5p, miR-200b-3p, miR-200b-5p, miR-200c-3p y miR-200c-5p [23]	216	0.997	99.03%	98.23%
miR-23a [24]	97	0.97	86%	100%
miR 28-5p [29]	12	0.937	85.5%	88.7%
miR-155 [7]	96	0.929	96%	89%
miR-125b-1-3p [27]	180	0.927	85.5%	80.1%
miR-200c-3p [23]	216	0.893	81.55%	83.19%
miR-200b [8]	97	0.880	75.58%	88.87%
miR-30b-3p [27]	180	0.878	84.3%	79.1%
miR-200a-5p [23]	216	0.876	78.64%	97.35%
miR-1226 [30]	122	0.87	85.7%	80.8%
miR-200a-3p [23]	216	0.865	77.67%	93.81%
miR-200b-5p [23]	216	0.864	74.76%	88.5%
miR-223 [8]	97	0.862	72.7%	89.3%
miR-3198 + miR-4299 [11]	61	0.86	68%	96%
miR-223-5p [26]	100	0.859	81%	78%
miR-200c-5p [23]	216	0.857	79.61%	79.65%
miR-200b-3p [23]	216	0.832	67.96%	87.61%
miR-199b-3p [19]	23	0.78	80%	72.7%
miR146a-5p [19]	23	0.74	72.7%	72.7%
miR-3198 [11]	61	0.72		
miR146a-5p [28]	210	0.702	66.7%	65.8%
miR-200b [3]	60	0.7	63%	79%
miR-145-5p [28]	210	0.621	56.7%	56.6%
miR-140-3p [28]	210	0.614	58.3%	57.9%
miR-429 [23]	216	0.567	35.92%	79.65%
miR-141-3p [23]	216	0.537	81.55%	34.51%
miR-141-5p [23]	216	0.508	25.24%	79.65%

miR: microRNA; AUC: area under the curve.

## Supplementary Materials

The following supporting information can be downloaded at: https://www.mdpi.com/article/10.3390/ijms25158274/s1.
