# Supplementary material for: Study of microRNAs in Gingival Crevicular Fluid as Periodontal Diseases Biomarkers: Systematic Review
Source: Int J Mol Sci. 2024 Jul 29;25(15):8274. doi: 10.3390/ijms25158274 (PMC11311819; doi:10.3390/ijms25158274)
Supplement: Supplementary file 1 [file ijms-25-08274-s001.zip › ijms-3105819-supplementary.pdf]

**SUPPLEMENTARY MATERIALS**

**Table S1.** MicroRNA expression levels in subjects with periodontitis vs. periodontal healthy subjects.

| | | miR expression level in case | | | miR expression level in control | | | p-valor |
|---|---|---|---|---|---|---|---|---|
| | Author | n | Mean | SD | n | Mean | SD | |
| **let-7e-5p** | Saito et al., (2017) | 6 | ΔΔ cq 0,6236 | 0,302 | 6 | ΔΔ cq -0,4249 | 0,4256 | **0.0006** |
| **let-7i-5p** | Saito et al., (2017) | 6 | ΔΔ cq -1,6596 | 0,374 | 6 | ΔΔ cq -2,25 | 0,1841 | 0,0060 |
| **miR-19a-3p** | Saito et al., (2017) | 6 | ΔΔ cq -2,3563 | 0,2335 | 6 | ΔΔ cq -1,2267 | 0,2457 | **<0.0001** |
| **miR-20a-3p** | Saito et al., (2017) | 6 | ΔΔ cq 3,4186 | 0,5381 | 6 | ΔΔ cq 4,0767 | 0,3768 | 0,0341 |
| **miR-20a-5p** | Saito et al., (2017) | 6 | ΔΔ cq -3,1824 | 0,3457 | 6 | ΔΔ cq -2,815 | 0,2255 | 0,0623 |
| **miR 21-3p** | Saito et al., (2017) | 6 | ΔΔ cq 2,922 | 0,8327 | 6 | ΔΔ cq 1,5316 | 0,3455 | **0.0036** |
| **miR-21c** | Liu et al., (2022) | 26 | 1.85 Pg/ml | 0.20 | 25 | 1.80 Pg/ml | 0.23 | 0,569 |
| **miR-22b** | Liu et al., (2022) | 26 | 1.78 Pg/ml | 0.32 | 25 | 1.82 Pg/ml | 0.35 | 0,978 |
| **miR-23a** | Liu et al., (2022) | 26 | 2.10 Pg/ml | 0.26 | 25 | 2.15 Pg/ml | 0.33 | 0,900 |
| | Zhang et al., (2019) | 29 | ΔΔct 6,12 | 0,24 | 21 | ΔΔct 2,92 | 0,26 | **<0.0001** |
| **miR-26b-5p** | Saito et al., (2017) | 6 | ΔΔ cq -2,333 | 0,2907 | 6 | ΔΔ cq -1,835 | 0,3151 | 0,0174 |
| **mir-27a** | Micó-Martínez et al., (2018) | 9 | 0,925 Pg/ml | 0,302 | 9 | 1,124 Pg/ml | 0,505 | 0,1963 |
| **miR-30a-5p** | Almiñana-Pastor et al., (2023) | 11 | 0,99 Pg/ml | 0,36 | 12 | 0,96 Pg/ml | 0,34 | n.s |
| **miR-30e-3p** | Saito et al., (2017) | 6 | ΔΔ cq 0,0153 | 0,199 | 6 | ΔΔ cq 0,441 | 0,2737 | 0,0116 |
| **miR-30e-5p** | Saito et al., (2017) | 6 | ΔΔ cq -2,2496 | 0,2483 | 6 | ΔΔ cq -1,3016 | 0,4058 | **0.0006** |
| **miR-31-3p** | Saito et al., (2017) | 6 | ΔΔ cq 0,552 | 0,3512 | 6 | ΔΔ cq -0,405 | 0,174 | **0.0001** |
| **miR-99a-5p** | Saito et al., (2017) | 6 | ΔΔ cq -0,8063 | 1,0469 | 6 | ΔΔ cq -2,2266 | 0,2022 | 0,0085 |
| **miR 100-5p** | Saito et al., (2017) | 6 | ΔΔ cq 3,1403 | 0,8065 | 6 | ΔΔ cq 0,9416 | 0,4518 | **0.0002** |
| **miR 101-3p** | Saito et al., (2017) | 6 | ΔΔ cq -2,2713 | 0,5071 | 6 | ΔΔ cq -1,2233 | 0,403 | **0.0027** |
| **miR-103** | Liu et al., (2022) | 26 | 2.24 Pg/ml | 0.35 | 25 | 2.01 Pg/ml | 0,32 | **<0.0001** |

| | | | | | | | | |
|---|---|---|---|---|---|---|---|---|
| **miR-106** | Liu et al., (2022) | 26 | 2.13 Pg/ml | 0.33 | 25 | 2.40 Pg/ml | 0.30 | **<0.0001** |
| **miR-125a-5p** | Saito et al., (2017) | 6 | ΔΔ cq -2,768 | 0,3523 | 6 | ΔΔ cq -3,6 | 0,2885 | **0.0012** |
| **miR-125b-5p** | Saito et al., (2017) | 6 | ΔΔ cq -2,608 | 0,4909 | 6 | ΔΔ cq -4,1433 | 0,3541 | **0.0001** |
| **miR-126-5p** | Saito et al., (2017) | 6 | ΔΔ cq 6,287 | 0,7167 | 6 | ΔΔ cq 6,9333 | 0,2872 | 0,0675 |
| **miR-140-5p** | Saito et al., (2017) | 6 | ΔΔ cq -1,548 | 0,3326 | 6 | ΔΔ cq -0,64 | 0,336 | **0.0008** |
| **miR-141** | Liu et al., (2022) | 26 | 1.36 Pg/ml | 0.22 | 25 | 1.32 Pg/ml | 0,3 | 0,441 |
| **miR-144-3p** | Saito et al., (2017) | 6 | ΔΔ cq 6,352 | 0,816 | 6 | ΔΔ cq 8,1016 | 1,7088 | 0,0191 |
| **miR-144-5p** | Saito et al., (2017) | 6 | ΔΔ cq 5,357 | 1,7088 | 6 | ΔΔ cq 7,0166 | 1,3842 | 0,0943 |
| **miR-145-5p** | Saito et al., (2017) | 6 | ΔΔ cq -2,873 | 0,5699 | 6 | ΔΔ cq -2,0233 | 0,307 | 0,0093 |
| **miR146a-5p,** | Almiñana-Pastor et al., (2023) | 11 | 2,06 Pg/ml | 1,43 | 12 | 1,03 Pg/ml | 0,8 | 0,05 |
| **miR-146a** | Radović et al., (2018) | 24 | | | 24 | 2,368 Pg/ml | 0.761 | |
| **miR-155** | Radović et al., (2018) | 24 | | | 24 | 2,179 Pg/ml | 0,785 | |
| **miR-155-5p** | Saito et al., (2017) | 6 | ΔΔ cq 0,4436 | 0,5213 | 6 | ΔΔ cq -1,3266 | 0,3522 | **<0.0001** |
| **miR-158** | Liu et al., (2022) | 26 | 2,3 Pg/ml | 0,33 | 25 | 2,29 Pg/ml | 0,33 | 0,628 |
| **miR-181b-5p** | Saito et al., (2017) | 6 | ΔΔ cq 1,712 | 0,2965 | 6 | ΔΔ cq 0,1983 | 0,3493 | **<0.0001** |
| **miR-187-3p** | Saito et al., (2017) | 6 | ΔΔ cq 2,972 | 0,3808 | 6 | ΔΔ cq 1,9883 | 0,3859 | 0,0012 |
| **miR-194-5p** | Saito et al., (2017) | 6 | ΔΔ cq 0,367 | 0,3495 | 6 | ΔΔ cq 0,9833 | 0,3058 | 0,0087 |
| **miR-199b-3p** | Almiñana-Pastor et al., (2023) | 11 | 2,09 Pg/ml | 1,59 | 12 | 0,93 Pg/ml | 0,31 | 0,03 |
| **miR-200a-5p** | Saito et al., (2017) | 6 | ΔΔ cq 4,7836 | 0,6204 | 6 | ΔΔ cq 3,4533 | 0,2176 | 0,0006 |
| **miR-200b** | Liu et al., (2022) | 26 | 3,06 Pg/ml | 0,21 | 25 | 2,47 Pg/ml | 0,16 | **<0.0001** |
| **miR-200b-3p** | Saito et al., (2017) | 6 | ΔΔ cq -2,5246 | 0,4365 | 6 | ΔΔ cq - 3,6966 | 0,1006 | 0,0001 |
| **miR-200b-5p** | Saito et al., (2017) | 6 | ΔΔ cq 2,2936 | 0,8303 | 6 | ΔΔ cq 1,6116 | 0,2056 | 0,0794 |
| **miR-200c-3p** | Saito et al., (2017) | 6 | ΔΔ cq -4,303 | 0,3905 | 6 | ΔΔ cq -4,955 | 0,1543 | 0,0035 |
| **miR-200c-5p** | Saito et al., (2017) | 6 | ΔΔ cq 6,1253 | 0,2075 | 6 | ΔΔ cq 5,0433 | 0,5072 | 0,0007 |

| | | | | | | | | |
|---|---|---|---|---|---|---|---|---|
| **miR-201a** | Liu et al., (2022) | 26 | 1,17 Pg/ml | 0,18 | 25 | 1,21Pg/ml | 0,19 | 0,892 |
| **miR-203** | Liu et al., (2022) | 26 | 1,31 Pg/ml | 0,12 | 25 | 1,26 Pg/ml | 0,12 | 0,401 |
| | Saito et al., (2017) | 6 | ΔΔ cq -5,5146 | 1,2485 | 6 | ΔΔ cq -6,3583 | 0,366 | 0,1433 |
| **miR-205-5p** | Saito et al., (2017) | 6 | ΔΔ cq -7,0563 | 0,3484 | 6 | ΔΔ cq -7,9283 | 0,1739 | 0,0003 |
| **miR-210-3p** | Saito et al., (2017) | 6 | ΔΔ cq 0,702 | 0,3484 | 6 | ΔΔ cq -1,665 | 0,3594 | 0,2095 |
| **miR-222-3p** | Saito et al., (2017) | 6 | ΔΔ cq -2,6196 | 0,1506 | 6 | ΔΔ cq -3,0616 | 0,324 | 0,0127 |
| **miR-223-5p** | Micó-Martínez et al., (2018) | 9 | 0,997 Pg/ml | 0,358 | 9 | 1,059 Pg/ml | 0,339 | 0,3757 |
| **miR223-3p** | Liu et al., (2022) | 26 | 3.25 Pg/ml | 0.45 | 25 | 1,58 Pg/ml | 0,37 | **<0.0001** |
| | Saito et al., (2017) | 6 | ΔΔ cq -11,136 | 0,2297 | 6 | ΔΔ cq -10,3666 | 0,2667 | 0,0003 |
| **miR-301a-3p** | Saito et al., (2017) | 6 | ΔΔ cq 2,097 | 0,7642 | 6 | ΔΔ cq 3,3616 | 0,511 | 0,0071 |
| **miR-320a** | Saito et al., (2017) | 6 | ΔΔ cq -1,5913 | 0,3572 | 6 | ΔΔ cq -2,1766 | 0,2252 | 0,0068 |
| **miR-338-3p** | Saito et al., (2017) | 6 | ΔΔ cq -3,2496 | 0,1764 | 6 | ΔΔ cq -2,4166 | 0,4428 | 0,0016 |
| **miR-338-5p** | Almiñana-Pastor et al., (2023) | 11 | 0,91Pg/ml | 0,33 | 12 | 1,07 Pg/ml | 0,45 | n.s |
| **miR-374a-5p** | Saito et al., (2017) | 6 | ΔΔ cq 0,9036 | 0,5201 | 6 | ΔΔ cq 1,5816 | 0,4976 | 0,0437 |
| **miR-582-5p** | Saito et al., (2017) | 6 | ΔΔ cq -1,1846 | 0,3001 | 6 | ΔΔ cq -0,5283 | 0,4976 | 0,0244 |
| **miR-590-5p** | Saito et al., (2017) | 6 | ΔΔ cq 1,8436 | 0,5431 | 6 | ΔΔ cq 2,9733 | 0,3785 | 0,0019 |
| **miR-1226-5p** | Micó-Martínez et al., (2018) | 9 | 0,39 Pg/ml | 0,334 | 9 | 1,376 Pg/ml | 0,993 | 0,0004 |
| **miR-1306** | Micó-Martínez et al., (2018) | 9 | 0,972 Pg/ml | 0,445 | 9 | 1,273 Pg/ml | 0,939 | 0,3282 |

*miR: microRNA; SD: Standard derivation; ΔΔ cq: relative normalized expression; pg/ml: picogram per milliliter.*

**Table S2:** PRISMA 2020 checklist

| Section and Topic | Item # | Checklist item | Location where item is reported |
|---|---|---|---|
| **TITLE** | | | |
| Title | 1 | Identify the report as a systematic review. | Page 1 |
| **ABSTRACT** | | | |
| Abstract | 2 | See the PRISMA 2020 for Abstracts checklist. | Page 1 |
| **INTRODUCTION** | | | |
| Rationale | 3 | Describe the rationale for the review in the context of existing knowledge. | Page 1-2 |
| Objectives | 4 | Provide an explicit statement of the objective(s) or question(s) the review addresses. | Page 2 |
| **METHODS** | | | |
| Eligibility criteria | 5 | Specify the inclusion and exclusion criteria for the review and how studies were grouped for the syntheses. | Page 14 |
| Information sources | 6 | Specify all databases, registers, websites, organisations, reference lists and other sources searched or consulted to identify studies. Specify the date when each source was last searched or consulted. | Page 14 |
| Search strategy | 7 | Present the full search strategies for all databases, registers and websites, including any filters and limits used. | Table S3 |
| Selection process | 8 | Specify the methods used to decide whether a study met the inclusion criteria of the review, including how many reviewers screened each record and each report retrieved, whether they worked independently, and if applicable, details of automation tools used in the process. | Page 14-15 |
| Data collection process | 9 | Specify the methods used to collect data from reports, including how many reviewers collected data from each report, whether they worked independently, any processes for obtaining or confirming data from study investigators, and if applicable, details of automation tools used in the process. | Page 14-15 |
| Data items | 10a | List and define all outcomes for which data were sought. Specify whether all results that were compatible with each outcome domain in each study were sought (e.g. for all measures, time points, analyses), and if not, the methods used to decide which results to collect. | Page 15 |
| | 10b | List and define all other variables for which data were sought (e.g. participant and intervention characteristics, funding sources). Describe any assumptions made about any missing or unclear information. | Page 15 |
| Study risk of bias assessment | 11 | Specify the methods used to assess risk of bias in the included studies, including details of the tool(s) used, how many reviewers assessed each study and whether they worked independently, and if applicable, details of automation tools used in the process. | Page 15 |
| Effect measures | 12 | Specify for each outcome the effect measure(s) (e.g. risk ratio, mean difference) used in the synthesis or presentation of results. | Page 15 |
| Synthesis | 13a | Describe the processes used to decide which studies were eligible for each synthesis (e.g. tabulating the study intervention characteristics and | not included |

| Section and Topic | Item # | Checklist item | Location where item is reported |
|---|---|---|---|
| methods | | comparing against the planned groups for each synthesis (item #5)). | |
| | 13b | Describe any methods required to prepare the data for presentation or synthesis, such as handling of missing summary statistics, or data conversions. | not included |
| | 13c | Describe any methods used to tabulate or visually display results of individual studies and syntheses. | not included |
| | 13d | Describe any methods used to synthesize results and provide a rationale for the choice(s). If meta-analysis was performed, describe the model(s), method(s) to identify the presence and extent of statistical heterogeneity, and software package(s) used. | not included |
| | 13e | Describe any methods used to explore possible causes of heterogeneity among study results (e.g. subgroup analysis, meta-regression). | not included |
| | 13f | Describe any sensitivity analyses conducted to assess robustness of the synthesized results. | not included |
| Reporting bias assessment | 14 | Describe any methods used to assess risk of bias due to missing results in a synthesis (arising from reporting biases). | not included |
| Certainty assessment | 15 | Describe any methods used to assess certainty (or confidence) in the body of evidence for an outcome. | not included |
| **RESULTS** | | | |
| Study selection | 16a | Describe the results of the search and selection process, from the number of records identified in the search to the number of studies included in the review, ideally using a flow diagram. | Page 3-4 |
| | 16b | Cite studies that might appear to meet the inclusion criteria, but which were excluded, and explain why they were excluded. | Page 3 |
| Study characteristics | 17 | Cite each included study and present its characteristics. | Page 5-7 |
| Risk of bias in studies | 18 | Present assessments of risk of bias for each included study. | Page 4 |
| Results of individual studies | 19 | For all outcomes, present, for each study: (a) summary statistics for each group (where appropriate) and (b) an effect estimate and its precision (e.g. confidence/credible interval), ideally using structured tables or plots. | Page 10-13 |
| Results of syntheses | 20a | For each synthesis, briefly summarise the characteristics and risk of bias among contributing studies. | not included |
| | 20b | Present results of all statistical syntheses conducted. If meta-analysis was done, present for each the summary estimate and its precision (e.g. confidence/credible interval) and measures of statistical heterogeneity. If comparing groups, describe the direction of the effect. | not included |
| | 20c | Present results of all investigations of possible causes of heterogeneity among study results. | not included |
| | 20d | Present results of all sensitivity analyses conducted to assess the robustness of the synthesized results. | not included |

| Section and Topic | Item # | Checklist item | Location where item is reported |
|---|---|---|---|
| Reporting biases | 21 | Present assessments of risk of bias due to missing results (arising from reporting biases) for each synthesis assessed. | Page 4 |
| Certainty of evidence | 22 | Present assessments of certainty (or confidence) in the body of evidence for each outcome assessed. | not included |
| **DISCUSSION** | | | |
| Discussion | 23a | Provide a general interpretation of the results in the context of other evidence. | Page 10-13 |
| | 23b | Discuss any limitations of the evidence included in the review. | not included |
| | 23c | Discuss any limitations of the review processes used. | Page 13 |
| | 23d | Discuss implications of the results for practice, policy, and future research. | Page 13-14 |
| **OTHER INFORMATION** | | | |
| Registration and protocol | 24a | Provide registration information for the review, including register name and registration number, or state that the review was not registered. | Page 14 |
| | 24b | Indicate where the review protocol can be accessed, or state that a protocol was not prepared. | Page 14 |
| | 24c | Describe and explain any amendments to information provided at registration or in the protocol. | no modifications were made |
| Support | 25 | Describe sources of financial or non-financial support for the review, and the role of the funders or sponsors in the review. | Page 15 |
| Competing interests | 26 | Declare any competing interests of review authors. | Page 15 |
| Availability of data, code and other materials | 27 | Report which of the following are publicly available and where they can be found: template data collection forms; data extracted from included studies; data used for all analyses; analytic code; any other materials used in the review. | Page 15 |

**Table S3:** Search strategy tailored to each database

| Database | Search equation used |
|---|---|
| **Pubmed** | (MicroRNAs[Title/Abstract] OR MicroRNA[Title/Abstract] OR miRNA[Title/Abstract] OR miRNAs[Title/Abstract] OR "Micro RNA" [Title/Abstract] OR "Micro RNAs" [Title/Abstract] OR "mi-RNA" [Title/Abstract] OR "mi-RNAs" [Title/Abstract] OR "Primary MicroRNA" [Title/Abstract] OR "Primary miRNA" [Title/Abstract] OR "pri-miRNA"[Title/Abstract] OR "pri miRNA" [Title/Abstract] OR "pre-miRNA" [Title/Abstract] OR "pre miRNA" [Title/Abstract] OR stRNA[Title/Abstract] OR "Small Temporal RNA"[Title/Abstract] OR MicroRNAs[MeSH]) AND ("Periodontal disease" [Title/Abstract] OR "Periodontal diseases" [Title/Abstract] OR Gingivitis[Title/Abstract] OR Periodontitis[Title/Abstract] OR Gingival[Title/Abstract] OR Periodontal[Title/Abstract] OR Parodontosis[Title/Abstract] OR Parodontoses[Title/Abstract] OR "Pyorrhea Alveolaris" [Title/Abstract] OR Periodontal diseases[MeSH] OR Gingivitis[MeSH] OR Periodontitis[MeSH]) |
| **Embase** | (MicroRNAs:ab,kw,ti OR MicroRNA:ab,kw,ti OR miRNA:ab,kw,ti OR miRNAs:ab,kw,ti OR "Micro RNA":ab,kw,ti OR "Micro RNAs":ab,kw,ti OR "mi-RNA":ab,kw,ti OR "mi-RNAs":ab,kw,ti OR "Primary MicroRNA":ab,kw,ti OR "Primary miRNA":ab,kw,ti OR "pri-miRNA":ab,kw,ti OR "pri miRNA":ab,kw,ti OR "pre-miRNA":ab,kw,ti OR "pre miRNA":ab,kw,ti OR stRNA:ab,kw,ti OR "Small Temporal RNA":ab,kw,ti OR "Micro RNA"/exp) AND ("Periodontal disease":ab,kw,ti OR "Periodontal diseases":ab,kw,ti OR Gingivitis:ab,kw,ti OR Periodontitis:ab,kw,ti OR Gingival:ab,kw,ti OR Periodontal:ab,kw,ti OR Parodontosis:ab,kw,ti OR Parodontoses:ab,kw,ti OR "Pyorrhea Alveolaris":ab,kw,ti OR Gingivitis/exp OR periodontitis/exp OR "Periodontal disease"/exp) |
| **Scopus** | ( TITLE-ABS-KEY ((micrornas OR microrna OR mirna OR mirnas OR "micro rna" OR "micro rnas" OR "m-rna" OR "m-rnas" OR "mi-rna" OR "mi-rnas" OR "primary microrna" OR "primary mirna" OR "pri-mirna" OR "pri mirna" OR "pre- |

| | |
|---|---|
| | mirna" OR "pre mirna" OR strna OR "small temporal rna" )) AND TITLE-ABS-KEY ( ( "periodontal disease" OR "periodontal diseases" OR gingivitis OR periodontitis OR gingival OR periodontal OR parodontosis OR parodontoses OR "pyorrhea alveolaris" ) ) ) |
| **Web of Science** | (TS=((MicroRNAs OR MicroRNA OR miRNA OR miRNAs OR "Micro RNA" OR "Micro RNAs" OR "mi-RNA" OR "mi-RNAs" OR "Primary MicroRNA" OR "Primary miRNA" OR "pri-miRNA" OR "pri miRNA" OR "pre-miRNA" OR "pre miRNA" OR stRNA OR "Small Temporal RNA") )) AND TS=(("Periodontal disease" OR "Periodontal diseases" OR Gingivitis OR Periodontitis OR Gingival OR Periodontal OR Parodontosis OR Parodontoses OR "Pyorrhea Alveolaris")) |
| **Cochrane** | (MicroRNAs OR MicroRNA OR miRNA OR miRNAs OR "Micro RNA" OR "Micro RNAs" OR "mi-RNA" OR "mi-RNAs" OR "Primary MicroRNA" OR "Primary miRNA" OR "pri-miRNA" OR "pri miRNA" OR "pre-miRNA" OR "pre miRNA" OR stRNA OR "Small Temporal RNA") AND ("Periodontal disease" OR "Periodontal diseases" OR Gingivitis OR Periodontitis OR Gingival OR Periodontal OR Parodontosis OR Parodontoses OR "Pyorrhea Alveolaris") in All Text |
| **Google schoolar** | allintitle: (MicroRNAs OR MicroRNA OR miRNA OR miRNAs OR "Micro RNA" OR "Micro RNAs" OR "mi-RNA" OR "mi-RNAs" OR "Primary miRNA" OR "pri-miRNA" OR "pri miRNA" OR "pre-miRNA" OR "pre miRNA" OR stRNA OR "Small Temporal RNA") AND ("Periodontal disease" OR "Periodontal diseases" OR Gingivitis OR Periodontitis OR Gingival OR Periodontal OR Parodontosis OR Parodontoses OR "Pyorrhea Alveolaris") |

**Table S4:** Excluded articles and reason for exclusion

| Article | Reason for exclusion |
|---|---|
| Bandi et al. | Almost identical title and same group of authors as another article |
| Han et al., (2021) | Review |
| Honda, (2009) | Experimental study in vitro |
| Laberge et al., (2023) | Review |
| Li et al., (2020) | To study the expression of microRNAs in samples other than gingival crevicular fluid |
| Venugopal et al., (2017) | To study the expression of microRNAs in samples other than gingival crevicular fluid |
| Venugopal et al., (2017) | To study the expression of microRNAs in samples other than gingival crevicular fluid |
| Xu et al., (2020) | Experimental study in vitro |
| Zhao et al., (2022) | Experimental study in vitro |
